# Pre-operative Neurocognitive Function Was More Susceptible to Decline in Isocitrate Dehydrogenase Wild-Type Subgroups of Lower-Grade Glioma Patients

**DOI:** 10.3389/fneur.2020.591615

**Published:** 2020-12-08

**Authors:** Zhe Zhang, Zeping Jin, Xiaojie Yang, Liang Zhang, Yang Zhang, Dayuan Liu, Xiaohan Chi, Shuyu Hao, Jie Feng, Nan Ji

**Affiliations:** ^1^Department of Neurosurgery, Beijing Tiantan Hospital, Capital Medical University, Beijing, China; ^2^National Clinical Research Center for Neurological Diseases (China), Beijing, China; ^3^Department of Psychiatry, Beijing Anding Hospital, Capital Medical University, Beijing, China; ^4^Department of Neurosurgery, The Second Affiliated Hospital of Hainan Medical University, Haikou, China; ^5^Beijing Neurosurgical Institute, Beijing Tiantan Hospital, Capital Medical University, Beijing, China; ^6^Beijing Cancer Institute, Beijing Institute for Brain Disorders, Capital Medical University, Beijing, China; ^7^Beijing Advanced Innovation Center for Big Data-Based Precision Medicine, Beihang University, Beijing, China

**Keywords:** lower-grade glioma, neuropsychology, neurocognitive function, isocitrate dehydrogenase (IDH) status, 1p19q co-deletion

## Abstract

**Background:** Neuropsychological deficits frequently occur in diffuse lower-grade glioma (DLGG) patients, but their relationship with molecular subgroups based on the 2016 World Health Organization (WHO) Classification of Tumors of the Central Nervous System (CNS) is unclear.

**Methods:** All patients enrolled for this study were divided into different subgroups according to the molecular-integrated 2016 CNS WHO and morphology-centric 2007 CNS WHO to compare their neurocognitive function (NCF) dysfunction. Univariate and multivariate analyses were used to assess the independent factors for NCF decline. The performance of NCF changes for discrimination of IDH and 1p19q status was evaluated by receiver operating characteristic (ROC).

**Results:** There was no significant difference in the clinical characteristics among the molecular and morphologic subgroups. In the molecular subgroups, significant differences in NCF alterations were found in terms of attention function, working memory and executive function in grade II glioma patients; in addition to these changes in NCF, memory function and abstract thinking were also significantly different in grade III glioma patients. The pairwise comparison further confirmed that patients with astrocytoma (A)/anaplastic astrocytoma (AA) with isocitrate dehydrogenase wild-type (IDHwt) glioma were more susceptible to severe cognitive decline in terms of the NCF performance described above. For the morphologic subgroups, only working memory was significantly different in grade III glioma patients. The distribution proportion was significantly different among each subgroup of DLGG (grade II, *P* = 0.001; grade III, *P* = 0.002). The proportion of extensive NCF decline (≥5 tests) was 4, 12, and 50% in the IDH mutant oligodendroglioma (IDHm-O), IDHm-A, and IDHwt-A subgroups, and this proportion was 33, 60, and 93% in the IDHm-AO, IDHm-AA, and IDHwt-AA subgroups, respectively. In multivariate regression analysis, molecular types were independent factors for NCF alterations after adjusted the factors of tumor and demographics (*p* < 0.05). ROC curves suggested combined NCF tests model showed an advantage in the differentiation of IDH status.

**Conclusions:** NCF alteration is closely related to molecular-integrated subgroups with varying degrees and frequencies in DLGG. Patients with IDHwt gliomas are more susceptible to suffer from severe and extensive NCF decline than other subgroups.

## Introduction

Diffuse lower-grade gliomas (DLGGs, World Health Organization grade II and grade III) are common infiltrative neoplasms in the central nervous system (CNS) of adults. Because of their aggressive and heterogeneous features, DLGGs will inevitably lead to neurological deficits, albeit with relatively slow growth (1, 2). According to reports, up to one-third of patients suffer from one or more deficits in neurocognitive domains, such as memory, attention, and executive function, which has a significant influence on quality of life (3–5). With the progression of medical technology, patients with DLGG tend to have prolonged survival, and their requirements are therefore even higher, being more concerned with neurocognitive function (NCF) than ever before (6, 7).

However, neurocognitive dysfunction is usually variable in diffuse glioma. In recent decades, several studies have focused on evaluating the influencing factors of neurocognitive dysfunction. Among many possible reasons, tumor grade and genetic alterations have been the most frequently investigated factors. Nevertheless, the relationship between NCF and tumor grade is still controversial (8). In contrast, existing studies have suggested that isocitrate dehydrogenase (IDH) status is a promising molecular marker closely related to NCF alteration in high-grade astrocytoma (grade III-IV) (9). Patients in the IDH wild-type (IDHwt) group were more susceptible to neurocognitive dysfunction than those in the IDH mutation (IDHm) group. A recent study further elaborated that IDHwt was a risk factor for neurocognitive dysfunction in diffuse glioma (grade II-IV) (3). These results illustrate that IDH status is a potent molecular marker for NCF subgrouping due to its ability to reflect the inherent characteristics of diffuse glioma. Consistently, the current classification of the CNS has recommended IDH status for reclassifying diffuse gliomas since 2016, and its clinical meaning for predicting treatment response and prognosis, especially for highly heterogeneous DLGGs, has been confirmed in both prior and subsequent studies (10). In light of the obvious advantages of the new classification, we hypothesize that NCF is characterized by IDH status before surgery in patients with DLGG as well.

However, to our knowledge, no prior study has particularly investigated the relationship between preoperative NCF and the subgroups of DLGG stratified by the 2016 World Health Organization (WHO) Classification of Tumors of the CNS, including IDHm and 1p19q co-deletion. In return, preoperative NCF might serve as an underlying clue for providing biological characteristics of diffuse glioma to some extent in the future.

Therefore, in the present study, we conducted a retrospective investigation on the difference in preoperative NCF between IDHm/1p19q co-deletion subgroups in DLGG. Moreover, we also analyzed the cognitive changes between subgroups based on morphologic features to determine whether molecular features are more correlated with neurocognitive dysfunction.

## Methods

### Patients

Patients suspected of having primary supratentorial DLGG on preoperative magnetic resonance imaging (MRI) and who received a preoperative NCF assessment in neurosurgical oncology 6 ward of Beijing Tiantan Hospital, Capital Medical University between December 2018 and January 2020 were considered for inclusion. The inclusion criteria were as follows: age ranging from 18 to 70 years; histological diagnosis of DLGG (WHO grade II and grade III) according to the 2016 CNS WHO; molecular testing of the status of IDH1 and chromosome 1p and 19q; preoperative Karnofsky Performance Score (KPS) >70; and no prior antitumor treatment. The exclusion criteria were as follows: suffering from other serious neurological or psychiatric diseases; unable to undergo NCF tests due to a premorbid intelligence quotient (IQ) score <85; auditory, visual, language, motor, or other serious cognitive problems; multiple lesions on preoperative MRI; and lack of pathological or molecular information. All participants were selected through similar and strict eligibility and exclusion criteria.

Their basic information and histological and molecular parameters (IDH1 mutations and chromosome 1p and 19q codeletion) were carefully reviewed and well-recorded according to their medical records by 2 neurosurgeons (DL and XC). Preoperative MRI sequences including T1 contrast-enhanced sequences and T2/fluid attenuation inversion recovery (FLAIR) sequences were used for analysis. The whole area of hyperintensity on T2-/FLAIR-weighted MRI scans was defined as tumor volume (9). All images were acquired on a 3T General Electric clinical scanner (Discovery MR750 using 32-channel phased array coils). All imaging features were reviewed and measured using Neurosoft PACS/RIS version 5.5 by two neuroradiologists (SJS and HCS) with over 10 years of experience. All doctors who performed data collection were blinded to the outcome of NCF. This study was approved by the Ethical Committee of Beijing Tiantan Hospital, Capital Medical University. All participants or their authorized relatives signed informed consent forms.

### Neuropsychological Test

All patients received a comprehensive NCF evaluation before surgery by a trained neuropsychology staff member under the supervision of a neuropsychologist. The comprehensive NCF evaluation protocol included: Intellectual functions, Wechsler Adult Intelligence Scale-3rd Edition (WAIS-III); Auditory short-term memory, Rey Auditory Verbal Learning Test (total learning) (RAVLT TL); Auditory long-term memory, Rey Auditory Verbal Learning Test (delayed recall) (RAVLT DR); Visual memory, Rey complex figure test-immediate recall (RCFT IR); Attention function, Trail making test (TMT) and Color trails test (CTT); Auditory working memory, WAIS-III Digit Span (forward & backward) (DS); Spatial working memory, WAIS-III Spatial Span (forward & backward) (SS); Categorical verbal fluency, Animal naming test (ANT); Selective attention and cognitive flexibility, Stroop test-time and accuracy (ST-T and ST-A) and Abstract thinking, WAIS-III Similarities (SI) (Table 1) (11–14). The clinical characteristics of well-matched normal individuals who received the same measurements described above were used to standardize the NCF test scores, which were converted into z-scores (mean = 0, standard deviation = 1, Supplementary Table 1). Additionally, z-scores of the NCF tests <-1 were considered to represent a decline in the related domains of NCF (15).

**Table 1:** Neuropsychological functions and tests.

**Neuropsychological functions**	**Test**	**Abbreviations**
Intellectual functions	Wechsler adult intelligence scale-third edition	WAIS-III
**Memory functions**		
Auditory short-time memory	Rey auditory verbal learning test-total learning	RAVLT TL
Auditory long-term memory	Rey auditory verbal learning test-delayed recall	RAVLT DR
Visual memory	Rey complex figure test- immediate recall	RCFT IR
Attention function	Trail making test	TMT
	Color trails test	CTT
**Working memory**		
Auditory working memory	WAIS-III digit span (forward & backward)	DS
Spatial working memory	WAIS-III spatial span (forward & backward)	SS
**Executive functions**		
Categorical verbal fluency	Animal naming test	ANT
Selective attention & cognitive flexibility	Stroop test-time	ST-T
	Stroop test-accuracy	ST-A
Abstract thinking	WAIS-III Similarities	SI

### Statistical Analyses

All analyses were conducted using the statistical software R, version 3.6.2 (R Institute for Statistical Computing). The one-way analysis of variance (ANOVA) test was used for numeric variables of clinical characteristics and z-scores of NCF tests. In addition, pairwise comparisons of one-way ANOVA tests were also conducted to compare the z-scores among different subgroups. The chi-square test was used for categorical variables. The proportion of patients who had NCF decline in each subgroup was analyzed by the Kruskal–Wallis test. Univariate and multivariate logistic regression analyses were used to assess the factors related to molecular alterations as dependent variables. Performance of NCF changes for discrimination of IDH and 1p19q status was analyzed by area under curve (AUC), positive predictive value (PPV), and negative predictive value (NPV) using receiver operating characteristic (ROC) curves. The maximum Youden's index (YI) value was selected as the best threshold. All tests were double-sided, and a *P* < 0.05 was considered statistically significant.

## Results

### Demographic and Clinical Characteristics

**Figure 1:**
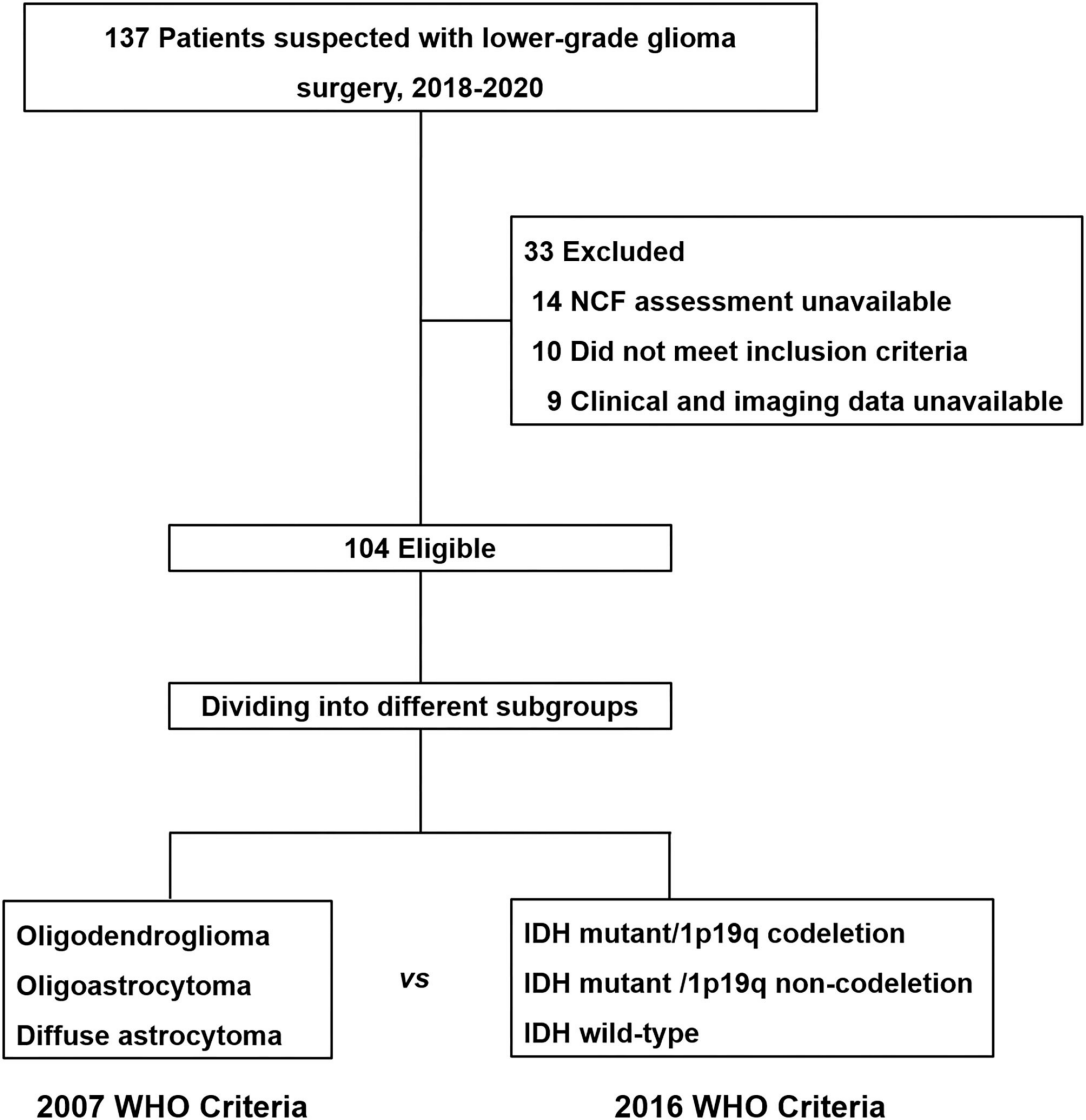
Flow diagram for neurocognitive function assessment cohort. All patients enrolled for this study were divided into different subgroups according to the molecular-integrated 2016 CNS WHO and morphology-centric 2007 CNS WHO to compare their neurocognitive function dysfunction.

In our study, a total of 104 patients (56 males and 48 females) who met the inclusion criteria were enrolled for the observation point of the study (Figure 1). The demographic and clinical characteristics of all patients are summarized in Table 2. All patients were divided into different subgroups according to molecular-integrated 2016 CNS WHO and morphology-centric 2007 CNS WHO. Tables 3, 4 summarize the clinical characteristics of the different molecular and morphologic subgroups, respectively. The results showed no differences in the clinical characteristics among these groups (molecular subgroups: age, *P* = 0.082; sex, *P* = 0.072; years of education, *P* = 0.480; seizure, *P* = 0.611; hemisphere, *P* = 0.217; tumor region, *P* = 0.302; lesion volume, *P* = 0.051; preoperative KPS, *P* = 0.056; and morphologic subgroups: age, *P* = 0.311; sex, *P* = 0.139; years of education, *P* = 0.274; seizure, *P* = 0.257; hemisphere, *P* = 0.193; tumor region, *P* = 0.989; lesion volume, *P* = 0.215; preoperative KPS, *P* = 0.992).

**Table 2:** Demographic and clinical characteristics.

**Characteristic**	***n* = 104**
**Age, y**	
Mean (SD), range	43.8 (10.7), 18–63
Male, *n* (%)	56 (54)
**Education, y**	
Mean (SD), range	10.8 (3.8), 6–16
**Molecular alteration (*****n*****, %)**	
**Grade II**	
IDHm-O	27 (26.0)
IDHm-A	25 (24.0)
IDHwt-A	10 (9.6)
**Grade III**	
IDHm-AO	18 (17.3)
IDHm-AA	10 (9.6)
IDHwt-AA	14 (13.5)
**Histology (*****n*****, %)**	
**Grade II**	
O	13 (12.5)
OA	27 (26.0)
A	22 (21.2)
**Grade III**	
AO	19 (18.3)
AOA	8 (7.7)
AA	15 (14.4)
Seizure history, yes (n, %)	41 (39.4)
**Hemisphere**	
Left (*n*, %)	60 (57.7)
**Region (*****n*****, %)**	
Frontal	60 (57.7)
Temporal	24 (23.1)
Parietal	13 (12.5)
Insular	4 (3.8)
Occipital	3 (2.9)
**Lesion volume, cm**^**3**^	
Mean (SD)	31.18 (26.82)
**KPS score**	
Median, range	90, 80–100

*IDHm-O, Oligodendroglioma, IDH mutant; IDHm-A, Astrocytoma, IDH mutant; IDHwt-A, Astrocytoma-IDH, wild-type; IDHm-AO, Anaplastic Oligodendroglioma, IDH mutant; IDHm-AA, Anaplastic Astrocytoma, IDH mutant; IDHwt-AA, Anaplastic Astrocytoma, IDH wild-type; O, Oligodendroglioma; A, Astrocytoma; OA, Oligoastrocytoma; AO, Anaplastic Oligodendroglioma; AA, Anaplastic Astrocytoma; AOA, Anaplastic Oligoastrocytoma; KPS, Karnofsky performance Score*.

**Table 3:** Clinical characteristics of molecular subgroups.

**Characteristic**	**WHO II (*****n*** **=** **62)**			**WHO III (*****n*** **=** **42)**			***P*-value**
	**IDHm-O (*n* = 27)**	**IDHm-A (*n* = 25)**	**IDHwt-A (*n* = 10)**	**IDHm-AO (*n* = 18)**	**IDHm-AA (*n* = 10)**	**IDHwt-AA (*n* = 14)**	
**Age, y**							
Mean (SD)	43.93 (9.56)	41.44 (10.78)	41.30 (10.60)	47.11 (11.33)	38.80 (8.38)	49.50 (11.43)	0.082
Male *n* (%)	18 (66.67)	12 (48.00)	3 (30.00)	6 (33.33)	7 (70.00)	10 (71.43)	0.072
**Education, y**							
Mean (SD)	10.15 (3.82)	11.08 (4.17)	12.50 (4.20)	11.56 (3.57)	9.90 (3.51)	10.21 (3.19)	0.480
Seizure history							0.611
Yes (*N*, %)	10 (37.04)	8 (32.00)	3 (30.00)	9 (50.00)	6 (60.00)	5 (35.71)	
Hemisphere							0.217
Left (N, %)	18 (66.67)	16 (64.00)	4 (40.00)	9 (50.00)	3 (30.00)	10 (71.43)	
Region (*N*, %)							0.302
Frontal	20 (74.07)	14 (56.00)	4 (40.00)	10 (55.56)	7 (70.00)	5 (35.71)	
Temporal	3 (11.11)	7 (28.00)	4 (40.00)	4 (22.22)	2 (20.00)	4 (28.57)	
Parietal	2 (7.41)	3 (12.00)	1 (10.00)	3 (16.67)	1 (10.00)	3 (21.43)	
Insular	1 (3.70)	1 (4.00)	1 (10.00)	1 (5.56)	0 (0)	0 (0)	
Occipital	1 (3.70)	0 (0)	0 (0)	0 (0)	0 (0)	2 (14.29)	
**Lesion volume, cm**^**3**^							
Mean (SD)	28.29 (24.03)	34.67 (28.12)	15.50 (14.35)	74.41 (36.75)	45.05 (39.20)	20.44 (20.94)	0.051
**KPS score**							
Median	90	90	90	90	90	90	0.056

*IDHm-O, Oligodendroglioma, IDH mutant; IDHm-A, Astrocytoma, IDH mutant; IDHwt-A, Astrocytoma, IDH wild-type; IDHm-AO, Anaplastic Oligodendroglioma, IDH mutant; IDHm-AA, Anaplastic Astrocytoma, IDH mutant; IDHwt-AA, Anaplastic Astrocytoma, IDH wild-type; KPS, Karnofsky performance Score*.

**Table 4:** Clinical characteristics of morphologic subgroups.

**Characteristic**	**WHO II (*****n*** **=** **62)**			**WHO III (*****n*** **=** **42)**			***P*-value**
	**O (*n* = 13)**	**OA (*n* = 27)**	**A (*n* = 22)**	**AO (*n* = 15)**	**AOA (*n* = 8)**	**AA (*n* = 19)**	
**Age, y**							
Mean (SD)	44.85 (10.42)	42.96 (9.82)	40.55 (10.48)	46.63 (11.71)	49.50 (12.08)	43.13 (10.36)	0.311
Male *n* (%)	6 (46.15)	17 (62.69)	10 (45.45)	7 (36.84)	4 (50.00)	12 (80.00)	0.139
**Education, y**							
Mean (SD)	9.54 (4.18),	10.89 (3.83)	11.73 (4.19)	11.32 (3.32)	12.00 (3.66)	9.27 (3.15)	0.274
Seizure history							0.257
Yes (*N*, %)	4 (30.77)	9 (33.33)	8 (36.36)	8 (53.33)	6 (75.00)	6 (31.57)	
Hemisphere							0.193
Left (*N*, %)	7 (53.85)	21 (77.78)	10 (45.45)	10 (52.63)	5 (62.50)	7 (46.67)	
Region (*N*, %)							0.989
Frontal	8 (61.54)	18 (66.67)	12 (54.55)	10 (52.63)	5 (62.50)	7 (46.67)	
Temporal	2 (15.38)	6 (22.22)	6 (27.27)	4 (21.05)	2 (25.00)	4 (26.67)	
Parietal	2 (15.38)	1 (3.70)	3 (13.64)	3 (15.79)	3 (15.79)	3 (20.00)	
Insular	1 (7.69)	1 (3.70)	1 (4.55)	1 (5.29)	0 (0)	0 (0)	
Occipital	0 (0)	1 (3.70)	0 (0)	1 (5.26)	0 (0)	1 (6.67)	
**Lesion volume, cm**^**3**^							
Mean (SD)	32.39 (27.20)	25.16 (20.80)	31.15 (29.04)	43.34 (25.58)	22.18 (18.16)	27.03 (28.86)	0.215
**KPS score**							
Median	90	90	90	90	90	90	0.992

*O, Oligodendroglioma; OA, Oligoastrocytoma; A, Astrocytoma; AO, Anaplastic Oligodendroglioma; AOA, Anaplastic Oligoastrocytoma; AA, Anaplastic Astrocytoma; KPS, Karnofsky performance Score*.

### Degree of NCF Alteration in DLGG

The results of NCF performances of each subgroup according to IDH status and 1p19q deletion are summarized in Table 5. The NCF performances of patients with grade II glioma among molecular-integrated subgroups showed significant differences in attention function (CTT, *F* = 7.187, *P* = 0.002), working memory (DS, *F* = 6.449, *P* = 0.003; SS *F* = 7.912, *P* < 0.001), and executive function (ANT, *F* = 4.858, *P* = 0.011).

**Table 5:** Differences of neurocognitive performances (z-scores) in subgroups based on molecule.

**NCF domain and test**	**z-score M (SD) of grade II Subgroups**			***F*-value**	***P*-value**	**z-score M (SD) of grade III Subgroups**			***F*-value**	***P*-value**
	**IDHm-O**	**IDHm-A**	**IDHwt-A**			**IDHm-AO**	**IDHm-AA**	**IDHwt-AA**		
**Memory functions**										
RAVLT TL	0.75 (1.54)	0.34 (1.00)	−0.25 (1.88)	1.911	0.157	−0.58 (1.76)	−1.60 (1.37)	−2.35 (1.65)	4.687	0.015
RAVLT DR	0.66 (0.82)	0.52 (1.02)	0.06 (1.34)	1.322	0.274	0.01 (1.36)	−0.77 (0.82)	−1.22 (1.42)	3.788	0.031
RCFT IR	0.46 (1.49)	−0.25 (1.42)	−0.13 (1.39)	1.712	0.189	−0.59 (1.06)	−1.23 (0.87)	−2.06 (0.64)	10.72	<0.001
**Attention function**										
TMT	0.20 (0.92)	−0.22 (0.86)	−0.46 (1.14)	2.278	0.111	−0.18 (1.04)	−0.46 (0.92)	−1.50 (1.28)	5.961	0.006
CTT	−0.12 (0.71)	−0.19 (0.61)	−1.04 (0.74)	7.187	0.002	−0.13 (0.94)	−0.25 (1.25)	−1.22 (1.06)	4.649	0.016
**Working memory**										
DS	0.56 (1.13)	0.08 (0.79)	−0.76 (1.15)	6.449	0.003	0.56 (1.90)	−0.83 (1.67)	−2.45 (1.38)	12.57	<0.001
SS	0.55 (1.24)	−0.14 (1.10)	−1.11 (1.03)	7.912	<0.001	0.34 (1.11)	−0.58 (1.19)	−1.65 (0.94)	13.37	<0.001
**Executive functions**										
ANT	0.17 (0.96)	−0.22 (1.36)	−1.20 (1.29)	4.858	0.011	−0.48 (1.14)	−0.61 (0.89)	−1.90 (0.67)	9.821	<0.001
ST-T	0.53 (1.01)	0.33 (0.83)	0.34 (1.03)	0.359	0.700	−0.96 (2.67)	−1.61 (1.47)	−2.75 (2.44)	2.254	0.118
ST-A	0.51 (1.86)	0.54 (1.57)	0.52 (1.99)	0.003	0.997	−1.59 (2.64)	−0.14 (1.82)	−5.64 (3.79)	12.10	<0.001
**Abstract thinking**										
SI	−0.20 (0.52)	−0.49 (0.46)	−0.52 (0.83)	2.291	0.110	−0.73 (0.40)	−1.08 (0.59)	−1.83 (0.41)	23.26	<0.001

*IDHm-O, Oligodendroglioma, IDH mutant; IDHm-A, Astrocytoma, IDH mutant; IDHwt-A, Astrocytoma, IDH wild-type; IDHm-AO, Anaplastic Oligodendroglioma, IDH mutant; IDHm-AA, Anaplastic Astrocytoma, IDH mutant; IDHwt-AA, Anaplastic Astrocytoma, IDH wild-type*.

**Figure 2:**
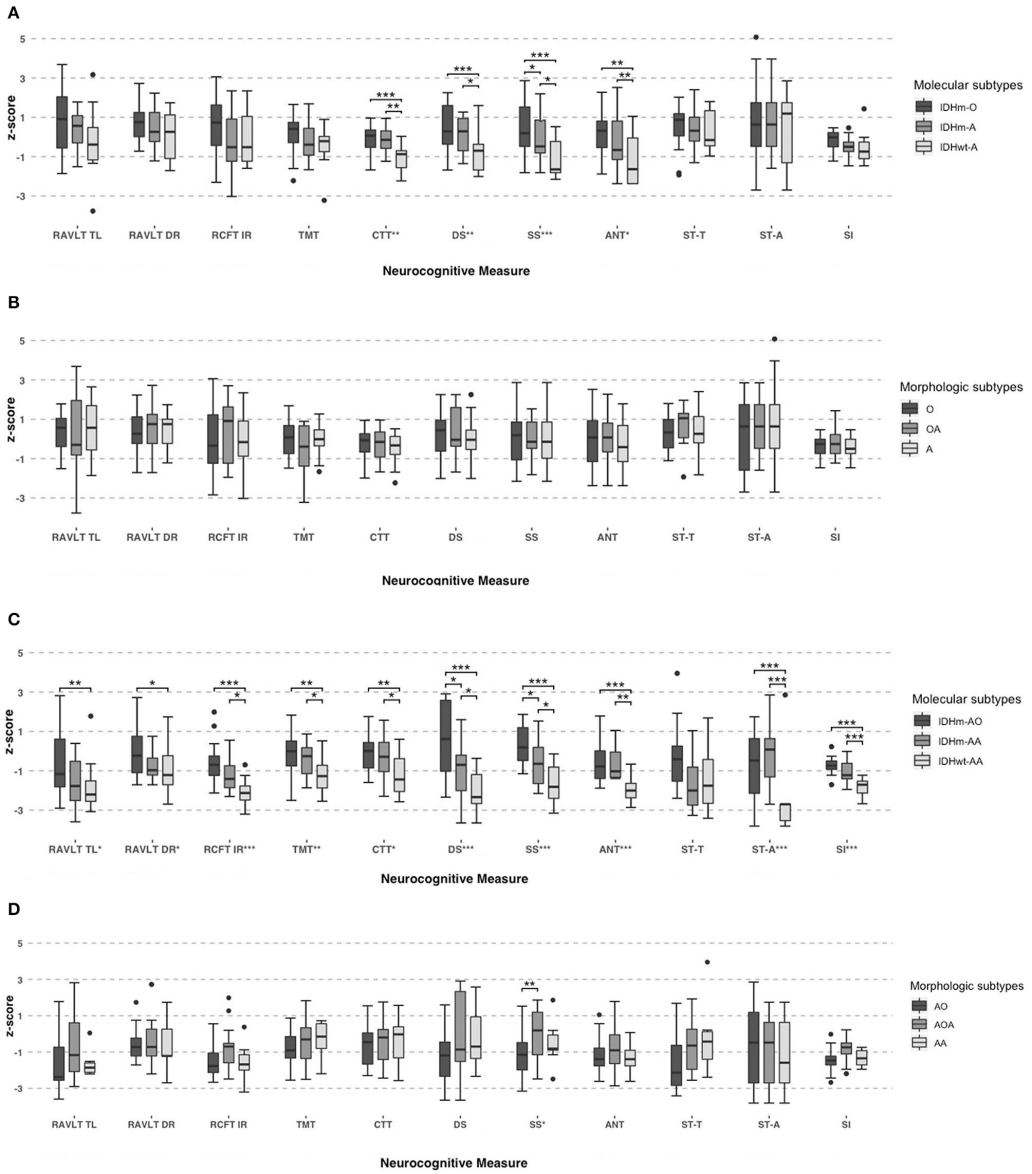
Neurocognitive performances in grade II subgroups based on molecular (IDH1 and 1p19q status) **(A)** and morphologic criteria **(B)**. And neurocognitive performances in grade III subgroups based on molecular (IDH1 and 1p19q status) **(C)** and morphologic criteria **(D)**. One-Way ANOVA tests and pairwise comparison used for group comparisons. *Significant, *P* < 0.05; **Significant, *P* < 0.01; ***Significant, *P* < 0.001.

Then, the pairwise comparison was used to analyze the NCF performances among the oligodendroglioma IDH-mutant (IDHm-O), astrocytoma IDH-mutant (IDHm-A) and astrocytoma IDH-wildtype (IDHwt-A) subgroups in patients with grade II glioma. There was a statistically significant difference between the IDHm-O and IDHwt-A subgroups in terms of attention function (CTT, *P* < 0.001), working memory (DS, *P* < 0.001; SS, *P* < 0.001) and executive function (ANT, *P* = 0.003). In addition, statistically significant differences were found between the IDHm-A and IDHwt-A subgroups in terms of attention function (CTT, *P* = 0.002), working memory (DS, *P* = 0.030; SS, *P* = 0.029), and executive function (ANT, *P* = 0.032). Moreover, only working memory showed a significant difference between the IDHm-O and IDHm-A subgroups (SS, *P* = 0.034). In the SS test, IDHm-O was the most favorable of all subgroups. The other tests for NCF did not show significant differences among the molecular subgroups. The pairwise comparison also demonstrated that the IDHwt-A subgroup had worse NCF than the IDHm-O and IDHm-A subgroups in terms of the mentioned tests (Figure 2A, Table 5).

Similarly, we also found significant differences among the grade III molecular subgroups in terms of memory function (RAVLT TL, *F* = 4.687, *P* = 0.015; RAVLT DR, *F* = 3.788, *P* = 0.031; RCFT IR, *F* = 10.72, *P* < 0.001), attention function (TMT, *F* = 5.961, *P* = 0.006; CTT, *F* = 4.649, *P* = 0.016), working memory (DS, *F* = 12.57, *P* < 0.001; SS, *F* = 13.37, *P* < 0.001), executive function (ANT, *F* = 9.821, *P* < 0.001; ST-A, *F* = 12.10, *P* < 0.001) and abstract thinking (SI, *F* = 23.26, *P* < 0.001).

Furthermore, pairwise comparisons were conducted to determine the differences among the anaplastic oligodendroglioma IDH-mutant (IDHm-AO), anaplastic astrocytoma IDH-mutant (IDHm-AA), and anaplastic astrocytoma IDH-wildtype (IDHwt-AA) subgroups in patients with grade III glioma. There were significant differences between the IDHm-AO and IDHwt-AA subgroups in terms of memory function (RAVLT TL, *P* = 0.004; RAVLT DR, *P* = 0.010; RCFT IR, *P* < 0.001), attention function (TMT, *P* = 0.002; CTT, *P* = 0.006), working memory (DS, *P* < 0.001; SS, *P* < 0.001), executive function (ANT, *P* < 0.001; ST-A, *P* < 0.001) and abstract thinking (SI, *P* < 0.001). The differences were statistically significant between the IDHm-AA and IDHwt-AA subgroups in terms of memory function (RCFT IR, *P* = 0.031), attention function (TMT, *P* = 0.028; CTT, *P* = 0.032), working memory (DS, *P* = 0.025; SS, *P* = 0.022), executive function (ANT, *P* = 0.002; ST-A, *P* < 0.001) and abstract thinking (SI, *P* < 0.001). There were statistically significant differences between the IDHm-AO and IDHm-AA subgroups in terms of working memory (DS: *P* = 0.044; SS: *P* = 0.037). The IDHm-AO subgroups, in terms of both the DS and SS tests, had the most favorable NCF among all subgroups. However, there was no significant difference in the other domains of NCF tests among the molecular subgroups. The pairwise comparison showed that the IDHwt-AA subgroup had a worse NCF performance than the IDHm-AO and IDHm-AA subgroups in terms of the above tests (Figure 2C, Table 5).

We also analyzed the NCF performances among the subgroups based on histology (Table 6). Nevertheless, there was no significant difference among the grade II subgroups based on morphologic features across the measures in terms of memory function, attention function, executive function and abstract thinking (all tests, *P* > 0.05, Figure 2B, Table 6). We found significant differences among the grade III subgroups in terms of working memory (SS, *P* = 0.037). Except for the SS in the grade III subgroup, there was no significant difference in each test of NCF among the morphologic subgroups (all tests except SS, *P* > 0.05, Figure 2D, Table 6).

**Table 6:** Differences of neurocognitive performances (z-scores) in subgroups based on morphology.

**NCF domain and test**	**z-score M (SD) of grade II Subgroups**			***F*-value**	***P*-value**	**z-score M (SD) of grade III Subgroups**			***F*-value**	***P*-value**
	**O**	**OA**	**A**			**AO**	**AOA**	**AA**		
**Memory functions**										
RAVLT TL	0.35 (2.16)	0.55 (1.39)	0.31 (0.93)	0.195	0.823	−0.82 (1.93)	−2.31 (1.51)	−1.69 (1.54)	2.402	0.104
RAVLT DR	0.60 (1.11)	0.50 (0.87)	0.46 (1.12)	0.079	0.924	−0.34 (1.41)	−1.03 (1.86)	−0.66 (0.98)	0.760	0.474
RCFT IR	0.58 (1.59)	−0.07 (1.35)	−0.03 (1.52)	0.981	0.381	−0.84 (1.17)	−1.55 (1.04)	−1.57 (0.88)	2.464	0.098
**Attention function**										
TMT	−0.44 (1.32)	−0.01 (0.74)	0.06 (0.93)	1.263	0.290	−0.52 (1.15)	−0.86 (1.85)	−0.80 (0.96)	0.301	0.742
CTT	−0.26 (0.89)	−0.39 (0.70)	−0.21 (0.71)	0.367	0.695	−0.43 (1.08)	−0.40 (1.41)	−0.70 (1.15)	0.283	0.755
**Working memory**										
DS	0.31 (1.22)	−0.01 (1.00)	0.26 (1.15)	0.507	0.605	−0.25 (2.24)	−0.70 (2.28)	−1.49 (1.75)	1.478	0.241
SS	−0.07 (1.04)	−0.02 (1.27)	0.07 (1.45)	0.051	0.950	−0.02 (1.35)	−0.52 (1.24)	−1.21 (1.23)	3.604	0.037
**Executive functions**										
ANT	0.04 (1.21)	−0.39 (1.19)	−0.13 (1.40)	0.556	0.576	−0.67 (1.29)	−1.36 (0.82)	−1.18 (1.01)	1.419	0.254
ST-T	0.65 (1.03)	0.41 (0.99)	0.29 (0.81)	0.589	0.558	−1.56 (2.49)	−1.07 (3.10)	−2.25 (2.01)	0.672	0.517
ST-A	0.38 (1.58)	0.96 (1.84)	0.08 (1.64)	1.665	0.198	−2.12 (3.06)	−3.95 (4.62)	−2.48 (3.83)	0.718	0.494
**Abstract thinking**										
SI	−0.26 (0.73)	−0.39 (0.51)	−0.40 (0.55)	0.298	0.744	−0.92 (0.66)	−1.32 (0.46)	−1.43 (0.66)	3.942	0.059

*O, Oligodendroglioma; OA, Oligoastrocytoma; A, Astrocytoma; AO, Anaplastic Oligodendroglioma; AOA, Anaplastic Oligoastrocytoma; AA, Anaplastic Astrocytoma*.

### Frequency of NCF Alteration in DLGG

**Figure 3:**
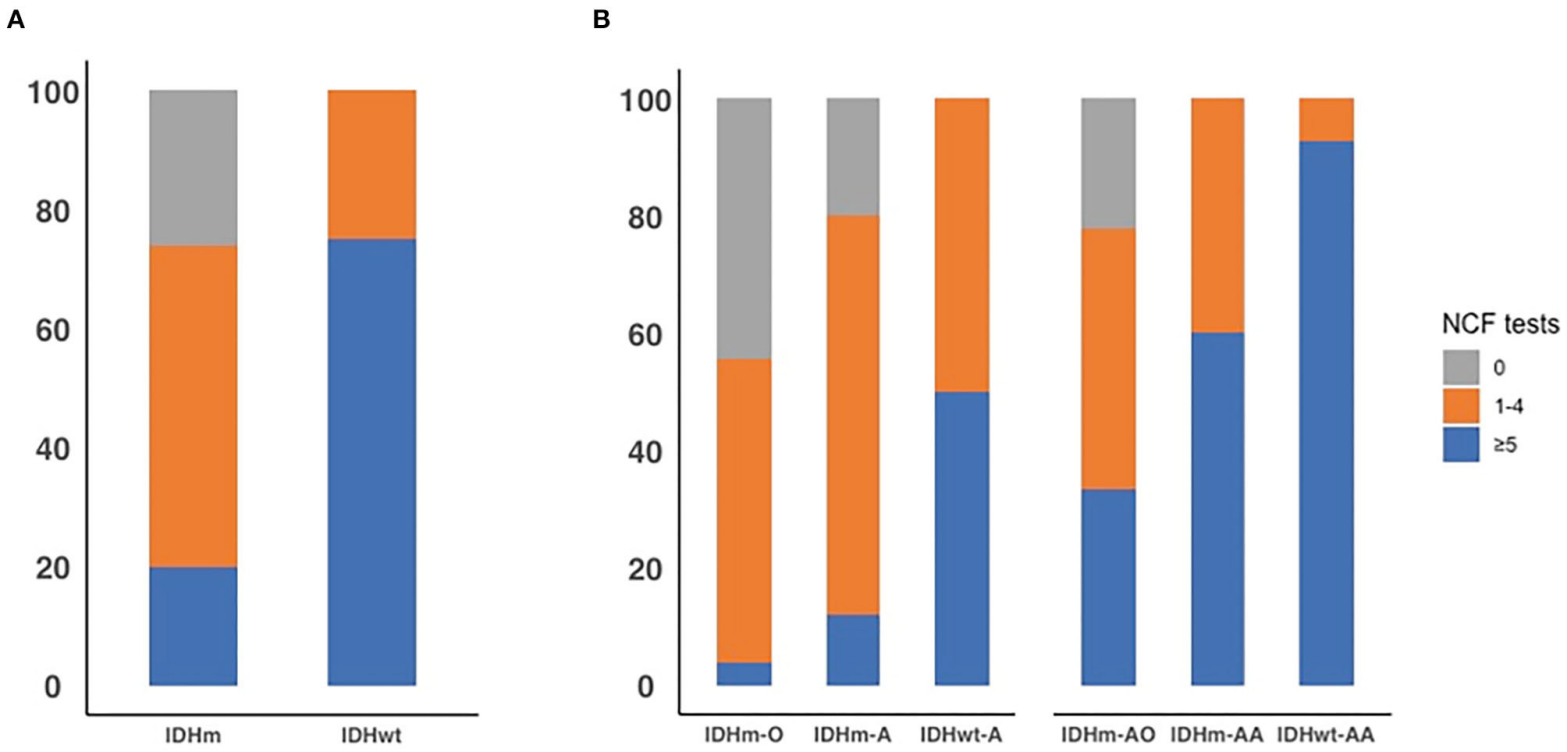
The proportion of all patients with diffusion lower-grade glioma with neurocognitive function decline across 0 test, 1 to 4 tests, and 5 or more tests by IDH status **(A)**. The proportion of all patients with diffusion lower-grade glioma with neurocognitive function decline across 0 test, 1 to 4 tests, and 5 or more tests in each subgroup based on IDH status and 1p19q deletion **(B)**.

We chose −1 as the cut-off value for the z-score across the NCF tests. The proportion of patients who had a NCF decline is shown in Figure 3 and Table 7. The proportion of NCF decline between the IDHm and IDHwt subgroups was significantly different (*P* < 0.001). The proportion of NCF decline (≥5 tests) in the IDHwt subgroup was higher than that in the IDHm subgroup in all lower-grade glioma patients (75 vs. 20%, Figure 3A). Additionally, in DLGG patients, the proportion of NCF decline among subgroups based on IDH status and 1p19q deletion was statistically significant (grade II, *P* = 0.001; grade III, *P* = 0.002, respectively). For grade II tumors, the proportion of patients without NCF decline was 44, 20, and 0% in the IDHm-O, IDHm-A, and IDHwt-A subgroups, while the proportion of patients with extensive NCF decline (≥5 tests) was 4, 12, and 50% in the IDHm-O, IDHm-A, and IDHwt-A subgroups, respectively. For grade III tumors, the proportion of patients without NCF decline was 22% in the IDHm-AO subgroup and 0% in the IDHm-AA and IDHwt-AA subgroups. In addition, the proportions of patients with extensive NCF decline (≥5 tests) were 33, 60, and 93% in the IDHm-AO, IDHm-AA, and IDHwt-AA subgroups, respectively (Figure 3B). The IDHwt-A/AA subgroup thus tended to have more extensive NCF decline than the other two subgroups within each grade, but the IDHm-O/AO subgroup showed the opposite trend.

**Table 7:** The proportion of NCF decline in molecular subgroups.

Number of NCF tests^*^	**Grade II (*****n*** **=** **62)**			***P*-value**	**Grade III (*****n*** **=** **42)**			***P*-value**
	**IDHm-O**	**IDHm-A**	**IDHwt-A**		**IDHm-AO**	**IDHm-AA**	**IDHwt-AA**	
0	12 (44%)	5 (20%)	0 (0%)	0.001	4 (22%)	0 (0%)	0 (0%)	0.002
1–4	14 (52%)	17 (68%)	5 (50%)		8 (44%)	4 (40%)	1 (7%)	
≥5	1 (4%)	3 (12%)	5 (50%)		6 (33%)	6 (60%)	13 (93%)	

*IDHm-A, Astrocytoma, IDH mutant; IDHwt-A, Astrocytoma, IDH wild-type; IDHm-AO, Anaplastic Oligodendroglioma, IDH mutant; IDHm-AA, Anaplastic Astrocytoma, IDH mutant; IDHwt-AA, Anaplastic Astrocytoma, IDH wild-type*.

* *The z-scores of NCF tests <-1 were considered to be a decline*.

### Univariate and Multivariate Analysis

Univariate analysis showed that age, KPS and molecular subtypes were significantly associated with NCF alteration for grade II. While, in grade III, the significant related factors for NCF alteration were age, location and molecular subtypes (*P* < 0.05). These variables were then subjected to the multivariate regression analysis. In multivariate regression analysis, we found that molecular types were independent factors for NCF alterations after adjusted the factors of tumor and demographics (*P* < 0.05). The results of the univariate and multivariate analysis were summarized in Tables 8, 9.

**Table 8:** Univariate analysis of clinical and molecular variables for lower-grade glioma patients.

**Variables**	**RAVLT TL**		**RAVLT DR**		**RCFT IR**		**TMT**		**CTT**		**DS**		**SS**		**ANT**		**ST-T**		**ST-A**		**SI**	
**Grade II**	**OR (95% CI)**	***P*****-value**	**OR (95% CI)**	***P*****-value**	**OR (95% CI)**	***P*****-value**	**OR (95% CI)**	***P*****-value**	**OR (95% CI)**	***P*****-value**	**OR (95% CI)**	***P*****-value**	**OR (95% CI)**	***P*****-value**	**OR (95% CI)**	***P*****-value**	**OR (95% CI)**	***P*****-value**	**OR (95% CI)**	***P*****-value**	**OR (95% CI)**	***P*****-value**
Age	1.038 (0.974–1.112)	0.269	1.014 (0.925–1.118)	0.763	1.095 (1.028–1.180)	0.009^**^	1.099 (1.018–1.208)	0.026^*^	0.966 (0.899–1.034)	0.324	0.969 (0.905–1.034)	0.35	1.041 (0.981–1.110)	0.195	0.980 (0.928–1.033)	0.449	1.062 (0.956–1.205)	0.293	1.045 (0.980–1.121)	0.197	0.995 (0.913–1.084)	0.898
Sex (Female, yes)	1.174 (0.325–4.240)	0.803	1.788 (0.276–14.366)	0.541	0.732 (0.229–2.246)	0.588	0.720 (0.167–2.818)	0.64	1.891 (0.483–8.156)	0.365	0.938 (0.242–3.500)	0.923	1.415 (0.439–4.658)	0.560	1.406 (0.488–4.098)	0.527	1.148 (0.131–10.103)	0.894	0.308 (0.063–1.170)	0.104	1.154 (0.199–6.708)	0.868
KPS <90	3.071 (0.679–12.930)	0.127	3.556 (0.424–24.607)	0.197	2.708 (0.676–10.636)	0.149	1.194 (0.162–5.833)	0.838	2.357 (0.439–10.668)	0.2778	3.592 (0.781–15.581)	0.087	5.600 (1.406–23.693)	0.015^*^	14.625 (3.242–105.146)	0.002^**^	1.600 (0.075–14.049)	0.697	5.238 (1.231–22.615)	0.023^*^	6.000 (0.968–37.932)	0.047^*^
**Location**																						
Frontal	2.400 (0.364–47.592)	0.437	–	0.994	3.214 (0.504–63.115)	0.296	1.688 (0.242–34.031)	0.647	0.750 (0.139–5.770)	0.751	0.438 (0.089–2.455)	0.314	0.622 (0.136–3.378)	0.551	0.724 (0.161–3.888)	0.682	–	0.996	0.903 (0.174–6.843)	0.909	–	0.994
Temporal	2.455 (0.261–54.513)	0.469	–	0.993	6.750 (0.884–142.452)	0.107	2.455 (0.261–54.513)	0.469	0.667 (0.068–6.505)	0.712	0.387 (0.043–2.898)	0.359	0.933 (0.155–6.016)	0.939	4.200 (0.788–27.353)	0.106	–	0.996	1.091 (0.146–9.789)	0.932	–	0.994
Other	Reference		Reference		Reference		Reference		Reference		Reference		Reference		Reference		Reference		Reference		Reference	
Tumor volume, cm^3^	1.013 (0.988–1.038)	0.305	1.011 (0.974–1.047)	0.519	1.004 (0.981–1.026)	0.719	1.003 (0.975–1.030)	0.799	0.998 (0.968–1.025)	0.885	1.009 (0.983–1.035)	0.478	0.996 (0.971–1.019)	0.744	1.017 (0.996–1.039)	0.112	1.020 (0.980–1.061)	0.316	1.020 (0.996–1.046)	0.105	0.998 (0.959–1.031)	0.891
**Molecular subtypes**																						
IDHm-1p19q–	Reference		Reference		Reference		Reference		Reference		Reference		Reference		Reference		Reference		Reference		Reference	
IDHm-1p19q+	1.095 (0.232–5.171)	0.906	–	0.996	1.361 (0.384–4.949)	0.631	2.000 (0.436–10.738)	0.381	1.091 (0.185–6.441)	0.920	1.091 (0.185–6.441)	0.920	2.526 (0.586–13.233)	0.229	7.385 (1.941–36.926)	0.006^**^	1.087 (0.122–9.670)	0.936	1.438 (0.336–6.514)	0.623	2.261 (0.204–50.549)	0.517
IDHwt	3.833 (0.723–21.218)	0.111	–	0.995	2.333 (0.470–11.285)	0.286	2.000 (0.232–14.322)	0.488	5.333 (0.944–34.056)	0.060	8.000 (1.500–51.313)	0.018^*^	12.000 (2.278–80.137)	0.005^**^	12.000 (2.278–80.137)	0.005^**^	–	0.996	2.464 (0.406–14.066)	0.304	11.143 (1.220–245.921)	0.05
**Grade III**																						
Age	1.163 (1.073–1.298)	0.001^**^	1.100 (1.030–1.192)	0.009^**^	1.132 (1.054–1.241)	0.002^**^	1.123 (1.044–1.232)	0.005^**^	1.019 (0.964–1.079)	0.516	1.082 (1.019–1.162)	0.017^*^	1.083 (1.018–1.165)	0.019^*^	1.067 (1.007–1.140)	0.038^*^	1.103 (1.034–1.194)	0.007^**^	1.089 (1.024–1.173)	0.012^*^	1.043 (0.986–1.108)	0.152
Sex (Female, yes)	1.800 (0.491–7.156)	0.383	0.758 (0.213–2.623)	0.663	0.593 (0.167–2.050)	0.410	0.464 (0.117–1.677)	0.252	0.423 (0.113–1.467)	0.183	0.714 (0.205–2.449)	0.592	0.423 (0.113–1.467)	0.183	0.388 (0.107–1.330)	0.138	0.733 (0.206–2.579)	0.627	0.884 (0.254–3.082)	0.845	0.467 (0.131–1.590)	0.228
**Grade II**	**OR (95% CI)**	***P*****-value**	**OR (95% CI)**	***P*****-value**	**OR (95% CI)**	***P*****-value**	**OR (95% CI)**	***P*****-value**	**OR (95% CI)**	***P*****-value**	**OR (95% CI)**	***P*****-value**	**OR (95% CI)**	***P*****-value**	**OR (95% CI)**	***P*****-value**	**OR (95% CI)**	***P*****-value**	**OR (95% CI)**	***P*****-value**	**OR (95% CI)**	***P*****-value**
KPS <90	2.037 (0.480–8.578)	0.325	1.3119 (0.317–5.361)	0.696	1.264 (0.312–5.669)	0.747	1.750 (0.416–7.257)	0.435	1.900 (0.472–7.975)	0.365	2.500 (0.596–13.084)	0.232	1.154 (0.278–4.658)	0.839	1.641 (0.408–7.346)	0.493	1.105 (0.271–4.973)	0.891	4.219 (0.903–30.735)	0.094	1.867 (0.465–8.367)	0.388
**Location**																						
Frontal	3.000 (0.557–16.805)	0.196	0.556 (0.113–2.500)	0.448	4.500 (0.887–25.396)	0.073	–	0.993	4.800 (0.939–36.936)	0.081	18.000 (3.171–156.656)	0.003^**^	5.778 (1.127–44.692)	0.052	3.214 (0.700–16.419)	0.140	–	0.994	–	0.997	7.933 (1.601–49.413)	0.016^*^
Temporal	0.444 (0.068–2.597)	0.374	0.074 (0.003–0.626)	0.035^*^	2.250 (0.027–1.661)	0.171	–	0.993	2.667 (0.383–24.345)	0.337	2.667 (0.383–24.345)	0.337	1.714 (0.220–16.091)	0.608	1.000 (0.161–1.192)	1.00	–	0.994	–	0.998	0.853 (0.062–4.542)	0.608
Other	Reference		Reference		Reference		Reference		Reference		Reference		Reference		Reference		Reference		Reference		Reference	
Tumor volume, cm^3^	1.011 (0.986–1.041)	0.426	1.004 (0.981–1.029)	0.718	0.933 (0.970–1.017)	0.572	1.006 (0.981–1.030)	0.638	0.994 (0.969–1.018)	0.629	1.001 (0.978–1.026)	0.930	1.001 (0.978–1.025)	0.913	0.990 (0.966–1.014)	0.417	0.999 (0.976–1.024)	0.961	1.004 (0.981–1.030)	0.741	0.998 (0.974–1.022)	0.853
**Molecular subtypes**																						
IDHm-1p19q–	Reference		Reference		Reference		Reference		Reference		Reference		Reference		Reference		Reference		Reference		Reference	
IDHm-1p19q+	1.867 (0.378–10906)	0.456	5.000 (0.909–32.783)	0.072	3.000 (0.623–16.112)	0.178	2.143 (0.325–14.415)	0.416	1.733 (0.327–9.128)	0.509	0.833 (0.163–3.992)	0.820	1.733 (0.327–9.128)	0.509	2.000 (0.410–10.160)	0.390	2.917 (0.598–17.170)	0.201	0.429 (0.073–2.097)	0.311	12.000 (1.969–107.893)	0.012^*^
IDHwt	2.933 (0.644–16.387)	0.182	9.000 (1.892–54.991)	0.009^*^	26.000 (3.829–532.912)	0.005^**^	9.000 (1.892–54.991)	0.009^**^	4.680 (1.094–22.949)	0.044^*^	7.500 (1.486–57.990)	0.025^*^	4.680 (1.094–22.949)	0.044^*^	12.000 (2.336–95.305)	0.006^**^	3.125 (0.739–15.120)	0.133	13.000 (1.944–262.191)	0.024^*^	–	0.994

*OR, odds ratio; CI, confidence interval; IDHm-1p19q-, IDH mutant and 1p19q-codeletion; IDHm-1p19q+, IDH mutant and IDHwt, 1p19q-noncodeletion; IDH wild-type*.

* significant P < 0.05;

** *significant P < 0.01*.

**Table 9:** Multivariate analysis of clinical and molecular variable in patients with lower-grade glioma.

**Variable**	**RCFT-IR**		**TMT**		**SS**		**ANT**		**ST-A**	
**Grade II**	**OR (95% CI)**	***P*****-value**	**OR (95% CI)**	***P*****-value**	**OR (95% CI)**	***P*****-value**	**OR (95% CI)**	***P*****-value**	**OR (95% CI)**	***P*****-value**
KPS <90							18.891 (3.209–195.684)	0.004^**^		
**Molecular subtypes**										
IDHm-1p19q-					Reference		Reference			
IDHm-1p19q+					2.423 (0.537–13.146)	0.263	10.640 (2.251–81.793)	0.007^**^		
IDHwt					9.230 (1.599–65.058)	0.016^*^	10.204 (1.396–100.833)	0.028^*^		
**Grade III**										
Age	1.345 (1.148–1.750)	0.004^**^	1.190 (1.069–1.401)	0.009^**^			1.084 (1.008–1.180)	0.040^*^	1.085 (1.008–1.186)	0.043^*^
Location										
Frontal										
Temporal										
Other										
**Molecular subtypes**										
IDHm-1p19q–	Reference		Reference				Reference		Reference	
IDHm-1p19q+	18.674 (6.836–41.969)	0.013^*^	16.477 (1.345–386.363)	0.044^*^			4.448 (0.727–34.432)	0.121	0.790 (0.120–4.964)	0.799
IDHwt	22.308 (2.468–63.901)	0.011^*^	19.131 (2.537–268.438)	0.011^*^			14.946 (2.482–153.789)	0.008^**^	15.761 (2.011–366.331)	0.025^*^

*OR, odds ratio; CI, confidence interval; IDHm-1p19q-, IDH mutant and 1p19q-codeletion; IDHm-1p19q+, IDH mutant and 1p19q-noncodeletion; IDHw, IDH wild-type*.

* significant P < 0.05;

** *significant P < 0.01*.

### ROC Curves of NCF Tests for Discrimination of IDH and 1p19q Status

In grade II, the AUCs of CTT (attention function) and SS (working memory) declines were 0.742 and 0.763 for identifying IDH status, respectively. While AUC of each NCF test for identification of 1p19q deletion status was <0.700. In grade III, the AUCs of RCFT IR (memory function), TMT (attention function), DS (working memory), ANT (executive functions), ST-A (executive functions), and SI (abstract thinking) for identifying IDH status were 0.750, 0.714, 0.714, 0.732, 0.768, and 0.819, respectively. In the aspect of districting 1p19q deletion status, only SI (abstract thinking) was with an AUC of 0.744, the other tests were with lower AUCs. Due to the low predictive efficiency of a single NCF test for IDH and 1p19q status, we further conducted a multivariate logistic regression analysis to examine the relationship between molecular alteration as a dependent variable and all NCF tests as independent variables. Then, a combined diagnostic model with multiple NCF tests was established based on the multivariate analysis (forward stepwise, *P* < 0.05) to predict molecular alterations. The AUC of the ROC curve was used to assess the performance of the combined NCF tests model in differentiating molecular alterations. In grade II, RAVLT DR, CTT and SS were independent factors related to IDH status. The combined NCF tests model [Logit(*P* | *y* = 1) = −4.117+3.664 × (RAVLT DR) +3.182 × (CTT) +2.852 × (SS)] allowed for further improvement in the differentiation of IDH status (ROC analysis: AUC = 0.842, 90.91% sensitivity, 68.63% specificity); in grade III, ST-A, and SI were independent factors associated with IDH status. The combined NCF tests model [Logit(*P* | *y* = 1) = −22.688+21.302 × (SI) +2.565 × (ST - A)] demonstrated an increasing ability to identify IDH status (ROC analysis: AUC = 0.918, 92.86% sensitivity, 85.71% specificity). However, ANT and SI were the independent factors associated with 1p19q deletion status for grade II and III, respectively. The combined NCF tests models for grade II [Logit(*P* | *y* = 1) = −0.613+1.999 × (ANT)] and grade III [Logit(*P* | *y* = 1) = −1.386+2.485 × (SI)] didn't show any improvement in the identification of 1p19q status. All the formulas for combined NCF tests models were summarized in detail in Supplementary Table 2. The performance of NCF tests for discriminating IDH and 1p19q status by ROC curves were shown in Supplementary Table 3.

## Discussion

To the best of our knowledge, this study is the first to represent neurocognitive dysfunction in molecular-integrated subgroups according to the 2016 CNS WHO classification for DLGG. Our data showed a wide range of NCF alterations among molecular groups classified by the status of IDH and 1p19q. Furthermore, we also compared the cognitive reduction in different molecular and morphologic groups. As our data show, patients in the IDHwt-A/AA subgroup were more susceptible to suffer from NCF dysfunction than patients in the IDHm-O/AO and IDHm-A/AA subgroups in terms of the severe and extensive NCF domains assessed for each grade of DLGG separately. However, these NCF alterations were hardly present in the morphologic subgroups. Thus, the molecular-integrated subgroups were more closely related to the severity and frequency of NCF reduction in DLGG with obvious superiority to the morphologic classification. Our results are consistent with a prior view on the relationship between NCF and IDH status. Wefel et al. illustrated that IDH status is valuable for varying NCF in a high-grade astrocytoma cohort (AA and GBM), and patients with IDHwt exhibited worse performances than those with IDHm (9). Recently, Kessel et al. further confirmed that IDH status plays an important role in the classification of NCF in diffuse grade II to IV glioma, but they did not analyze DLGG within each grade in detail (3). Notably, DLGG with high heterogeneity severely affected patients' quality of life and prognosis (6, 16, 17). Even more, unfortunately, studies focusing on a variety of preoperative NCF alterations by IDH and 1p19q status remain unavailable as far as we know. Thus, our study could be of high clinical meaning for clarifying the relationship between NCF and molecular subgroups.

Additionally, the 2016 CNS WHO introduced molecular markers, IDH mutation and 1p19q codeletion in addition to histology to identify biological entities of DLGG (18). As multitudinous studies have documented, these markers can assist clinicians in understanding tumor behaviors and are commonly used for predicting therapeutic efficacy and prognosis (19). Consistently, we have found that molecular alterations of IDH and 1p19q would serve as potent markers for identifying NCF dysfunction, although only a few tests of NCF have shown differences between 1p19q co-deletion and non-co-deletion. Thus, a large cohort would be more meaningful to further determine its cognitive value in the future.

In prior studies, tumor momentum was proposed to explain the relationship between invasiveness, tumor growth rate and neurological symptoms. It is believed that more invasive tumors have greater tumor momentum, and in return, more considerable tumor momentum could cause more severe symptoms (8, 9, 20). Our results subsequently confirmed this view and showed that IDHwt-A glioma represented an aggressive NCF decline compared with IDHm glioma. Genetically, IDHwt-A glioma exhibits more glioblastoma-like characteristics, such as a stronger angiogenesis ability and higher cell proliferation, compared with IDHm glioma (21–23). These inherent characteristics indicate that IDHwt-A glioma has higher invasiveness and greater tumor momentum than IDHm glioma. Furthermore, highly invasive IDHwt-A glioma not only damages the brain network more quickly but also has a negative effect on neuroplasticity, which ultimately leads to worse NCF performance (24–28). As our data suggest, patients who present with more rapid and extensive NCF decline may be likely to harbor aggressive characteristics, such as genetic alteration of IDHwt. And multivariate analysis further confirmed that molecular types were independent factors associated with working memory and executive function in grade II, and memory function, attention function, and executive function in grade III. Moreover, in ROC analysis, NCF test across one domain had limitations of sensitivity and specificity to stratify molecular subtypes, especially for 1p19q deletion status. Meanwhile, combined models of NCF tests showed an advantage in the differentiation of IDH status, but they were still unable to improve the ability to stratify 1p19q deletion status. Therefore, comprehensive assessment of NCF decline would enhance the understanding of tumor aggressive behavior and molecular subtypes, such as IDHwt, before surgery. For the aggressive tumors, early surgery and aggressive treatments should be performed to reduce damage to the normal brain.

## Limitation

There are still a few limitations in our study. As our results showed, only a few tests exhibited significant differences between the IDHm-O and IDHm-A subgroups. The insufficient sample size of our study is responsible for this limitation. Alternatively, perhaps the current neuropsychological assessment was unable to detect very small NCF alterations between these two groups. In view of this, it is necessary to employ a large cohort and to explore more sensitive and comprehensive tests for NCF evaluation in the future. Meanwhile, it also points out that our retrospective study cohort was from a single-center study, which will be inevitable to result in bias of analysis. Therefore, an extramural cohort or a multi-centric prospective study should be conducted to confirm the generalizability of our findings. Additionally, the present study did not investigate the mechanisms of IDH status causing NCF dysfunctions, such as microenvironments or special metabolites, which should be given more attention in further research. Besides, to get a uniform cohort for analysis of the relationship between molecular status and NCF changes, the IDH gene alteration of this study is only relative to IDH1 mutation. The patients with IDH 2 mutations were not enrolled for analysis. Given the great importance of IDH 2 gen alteration as well, we will enlarge our cohort and explore its relationship with NCF in the future.

## Conclusions

In summary, NCF alteration is closely related to molecular-integrated subgroups in DLGG. The present study first shows various frequencies and severities of NCF decline by IDH status and 1p19q deletion. According to neuropsychological assessments, patients with IDHwt-A/AA gliomas are more susceptible to suffer from severe and extensive NCF decline than patients with other subgroups of grade II and III gliomas, respectively.

## Data Availability Statement

The raw data supporting the conclusions of this article will be made available by the authors, without undue reservation.

## Ethics Statement

The studies involving human participants were reviewed and approved by the Ethical Committee of Beijing Tiantan Hospital, Capital Medical University. The patients/participants provided their written informed consent to participate in this study.

## Author Contributions

ZZ and ZJ: study conception, analysis, design, and manuscript drafting. XY and LZ: neurocognitive function evaluation. DL and XC: clinical information collection and acquisition of data. JF and SH: data analysis, manuscript preparation, and revising. NJ: study design, approved the final version of the manuscript and study supervision. All authors critically revised the article and reviewed the submitted version of the manuscript.

## Conflict of Interest

The authors declare that the research was conducted in the absence of any commercial or financial relationships that could be construed as a potential conflict of interest.
