# Supplementary material for: Pre-operative Neurocognitive Function Was More Susceptible to Decline in Isocitrate Dehydrogenase Wild-Type Subgroups of Lower-Grade Glioma Patients
Source: Front Neurol. 2020 Dec 8;11:591615. doi: 10.3389/fneur.2020.591615 (PMC7752952; doi:10.3389/fneur.2020.591615)
Supplement: Supplementary file 1 [file Table_1.docx]

Supplement table 1 Demographic and clinical characteristics of healthy control cohort

| Characteristic | n=30 |
| --- | --- |
| Age, y |  |
| Mean (SD), range | 43.4 (6.6), 25-56 |
| Male, n (%) | 15 (50) |
| Education, y |  |
| Mean (SD), range | 10.6 (3.9), 6-16 |
