# Supplementary material for: Pre-operative Neurocognitive Function Was More Susceptible to Decline in Isocitrate Dehydrogenase Wild-Type Subgroups of Lower-Grade Glioma Patients
Source: Front Neurol. 2020 Dec 8;11:591615. doi: 10.3389/fneur.2020.591615 (PMC7752952; doi:10.3389/fneur.2020.591615)
Supplement: Supplementary file 2 [file Table_2.docx]

Supplement Table 2 Summary the formulas of combined NCF tests models

|  | The combined NCF tests model for IDH status |
| --- | --- |
| Grade II | Logit(P \| y=1)= -4.117+3.664×(RAVLT DR)+3.182×(CTT)+2.852×(SS) |
|  | y: IDHwt=1; IDHm=0; RAVLT DR: normal=1, decline=0; CTT: normal=1, decline=0; SS: normal=1, decline=0 |
| Grade III | Logit(P \| y=1)= -22.688+21.302×(SI)+2.565×(ST-A) |
|  | y: IDHwt=1; IDHm=0; SI: normal=1, decline=0; ST-A: normal=1, decline=0 |
|  | The combined NCF tests model for 1p19q status |
| Grade II | Logit(P \| y=1)= -0.613+1.999×(ANT) |
|  | y: 1p19q non-codeletion=1; 1p19q codeletion=0; ANT: normal=1, decline=0 |
| Grade III | Logit(P \| y=1)= -1.386+2.485×(SI) |
|  | y: 1p19q non-codeletion=1; 1p19q codeletion=0; SI: normal=1, decline=0 |
