# Supplementary material for: Pre-operative Neurocognitive Function Was More Susceptible to Decline in Isocitrate Dehydrogenase Wild-Type Subgroups of Lower-Grade Glioma Patients
Source: Front Neurol. 2020 Dec 8;11:591615. doi: 10.3389/fneur.2020.591615 (PMC7752952; doi:10.3389/fneur.2020.591615)
Supplement: Supplementary file 3 [file Table_3.docx]

Supplement Table 3 ROC curves of neurocognitive function (NCF) tests for discrimination of IDH and 1p19q deletion status

|  | IDH (Grade II) | | | 1p19q (Grade II) | | | IDH (Grade III) | | | 1p19q (Grade III) | | |
| --- | --- | --- | --- | --- | --- | --- | --- | --- | --- | --- | --- | --- |
| Variable | AUC | SEN% | SPE% | AUC | SEN% | SPE% | AUC | SEN% | SPE% | AUC | SEN% | SPE% |
| RAVLT TL | 0.623 | 40.00 | 84.62 | 0.506 | 16.00 | 85.19 | 0.589 | 78.57 | 39.29 | 0.572 | 70.00 | 44.44 |
| RAVLT DR | 0.631 | 30.00 | 96.15 | 0.540 | 8.00 | 100.00 | 0.643 | 64.29 | 64.29 | 0.611 | 50.00 | 72.22 |
| RCFT IR | 0.575 | 40.00 | 75.00 | 0.529 | 28.00 | 77.78 | 0.750 | 92.86 | 57.14 | 0.572 | 70.00 | 44.44 |
| TMT | 0.523 | 20.00 | 84.62 | 0.544 | 20.00 | 88.89 | 0.714 | 64.29 | 78.57 | 0.567 | 30.00 | 83.33 |
| CTT | 0.742 | 60.00 | 88.46 | 0.504 | 12.00 | 88.89 | 0.661 | 64.29 | 67.86 | 0.561 | 40.00 | 72.22 |
| DS | 0.692 | 50.00 | 88.46 | 0.504 | 12.00 | 88.89 | 0.714 | 85.71 | 57.14 | 0.522 | 60.00 | 44.44 |
| SS | 0.763 | 70.00 | 82.69 | 0.564 | 24.00 | 88.89 | 0.679 | 64.29 | 71.43 | 0.589 | 40.00 | 77.78 |
| ANT | 0.656 | 60.00 | 71.15 | 0.684 | 48.00 | 88.89 | 0.732 | 85.71 | 60.71 | 0.583 | 50.00 | 66.67 |
| ST-T | 0.512 | 10.00 | 92.31 | 0.503 | 8.00 | 92.59 | 0.589 | 71.43 | 46.43 | 0.628 | 70.00 | 55.56 |
| ST-A | 0.563 | 30.00 | 82.69 | 0.526 | 20.00 | 85.19 | 0.768 | 92.86 | 60.71 | 0.572 | 70.00 | 44.44 |
| SI | 0.621 | 30.00 | 94.23 | 0.521 | 8.00 | 96.30 | 0.819 | 93.33 | 70.37 | 0.744 | 60.00 | 88.89 |
| cNCF | 0.842 | 90.91 | 68.63 | 0.684 | 48.00 | 88.89 | 0.918 | 92.86 | 85.71 | 0.744 | 60.00 | 88.89 |

Abbreviation: cNCF, combined NCF tests.
